# Association of Long-term Exposure to Ambient Air Pollutants With Risk Factors for Cardiovascular Disease in China

**DOI:** 10.1001/jamanetworkopen.2019.0318

**Published:** 2019-03-08

**Authors:** Bo-Yi Yang, Yuming Guo, Iana Markevych, Zhengmin (Min) Qian, Michael S. Bloom, Joachim Heinrich, Shyamali C. Dharmage, Craig A. Rolling, Savannah S. Jordan, Mika Komppula, Ari Leskinen, Gayan Bowatte, Shanshan Li, Gongbo Chen, Kang-Kang Liu, Xiao-Wen Zeng, Li-Wen Hu, Guang-Hui Dong

**Affiliations:** 1 Guangzhou Key Laboratory of Environmental Pollution and Health Risk Assessment, Guangdong Provincial Engineering Technology Research Center of Environmental and Health Risk Assessment, Department of Occupational and Environmental Health, School of Public Health, Sun Yat-sen University, Guangzhou, China; 2 Department of Epidemiology and Preventive Medicine, School of Public Health and Preventive Medicine, Monash University, Melbourne, Australia; 3 Institute of Epidemiology, Helmholtz Zentrum München–German Research Center for Environmental Health, Neuherberg, Germany; 4 Division of Metabolic and Nutritional Medicine, Dr von Hauner Children’s Hospital, Ludwig Maximilian University of Munich, Munich, Germany; 5 Institute and Clinic for Occupational, Social and Environmental Medicine, University Hospital of Munich, Munich, Germany; 6 Department of Epidemiology and Biostatistics, College for Public Health and Social Justice, Saint Louis University, St Louis, Missouri; 7 Department of Environmental Health Sciences, University at Albany, State University of New York, Rensselaer, New York; 8 Department of Epidemiology and Biostatics, University at Albany, State University of New York, Rensselaer, New York; 9 Comprehensive Pneumology Center Munich, German Center for Lung Research, Munich, Germany; 10 Allergy and Lung Health Unit, Centre for Epidemiology and Biostatistics, School of Population and Global Health, The University of Melbourne, Melbourne, Australia; 11 Murdoch Children Research Institute, Melbourne, Australia; 12 Finnish Meteorological Institute, Kuopio, Finland; 13 The National Institute of Fundamental Studies, Kandy, Sri Lanka

## Abstract

**Importance:**

Which cardiometabolic risk factors (eg, hypertension, type 2 diabetes, overweight or obesity, and dyslipidemia) are more sensitive to long-term exposure to ambient air pollution and whether participants with these conditions are more susceptible to the cardiovascular effects of air pollution remain unclear.

**Objectives:**

To evaluate the associations among long-term exposure to air pollutants, cardiometabolic risk factors, and cardiovascular disease (CVD) prevalence.

**Design, Setting, and Participants:**

This population-based cross-sectional study was conducted from April 1 through December 31, 2009, in 3 cities in Northeastern China. Participants were adults aged 18 to 74 years who had lived in study area for 5 years or longer. Data analysis was performed from May 1 through December 31, 2018.

**Exposures:**

Long-term (2006-2008) exposure to air pollutants was measured using a spatiotemporal statistical model (particulate matter with an aerodynamic diameter of ≤2.5 μm [PM_2.5_] and ≤1.0 μm [PM_1.0_]) and data from air monitoring stations (particulate matter with an aerodynamic diameter of ≤10.0 μm [PM_10.0_], sulfur dioxide [SO_2_], nitrogen dioxide [NO_2_], and ozone [O_3_]).

**Main Outcomes and Measures:**

Cardiovascular disease was determined by self-report of physician-diagnosed CVD. Blood pressure, body mass index, and levels of triglycerides and low-density lipoprotein cholesterol were measured using standard methods.

**Results:**

Participants included 15 477 adults (47.3% women) with a mean (SD) age of 45.0 (13.5) years. The prevalence of CVD was 4.8%, and the prevalence of cardiometabolic risk factors ranged from 8.6% (hyperbetalipoproteinemia) to 40.5% (overweight or obesity). Mean (SD) air pollutant concentrations ranged from 35.3 (5.5) μg/m^3^ (for NO_2_) to 123.1 (14.6) μg/m^3^ (for PM_10.0_). Associations with air pollutants were identified for individuals with hyperbetalipoproteinemia (eg, odds ratio [OR], 1.36 [95% CI, 1.03-1.78] for a 10-μg/m^3^ increase in PM_1.0_) and the weakest association for those with for overweight or obesity (eg, OR, 1.06 [95% CI, 1.02-1.09] for a 10-μg/m^3^ increase in PM_1.0_). Cardiometabolic risk factors only partially mediated associations between air pollution and CVD. However, they modified the associations such that greater associations were found in participants with these cardiometabolic conditions (eg, ORs for CVD and per 10-μg/m^3^ increase in PM_1.0_, 1.22 [95% CI, 1.12-1.33] in participants with hyperbetalipoproteinemia and 1.07 [95% CI, 0.98-1.16] in participants without hyperbetalipoproteinemia).

**Conclusions and Relevance:**

In this population-based study of Chinese adults with CVD, long-term exposure to air pollution was associated with a higher prevalence of cardiometabolic risk factors, and the strongest associations were observed for hyperbetalipoproteinemia. In addition, participants with cardiometabolic risk factors may have been more vulnerable to the effects of air pollution on CVD.

## Key Points

**Question:**

Which cardiometabolic risk factors are associated with ambient air pollution in China?

**Findings:**

In this population-based cross-sectional study including 15 477 Chinese adults, the strongest associations for exposure to air pollution were detected for individuals with hyperbetalipoproteinemia and the weakest associations for those with overweight or obesity. The metabolic risk factors may have exacerbated the associations of air pollutants with the prevalence of cardiovascular disease.

**Meaning:**

These findings may help researchers, physicians, and policy makers evaluate the hazardous effects of air pollutants more completely and design targeted strategies for primary prevention of cardiovascular disease.

## Teaser

This population-based cross-sectional study evaluates the associations of long-term exposures to air pollutants with cardiometabolic risk factors and prevalence of cardiovascular disease among adults in China.

## Introduction

Exposure to air pollution is associated with increased cardiovascular mortality and morbidity.^1,2^ The proposed mechanisms include systemic inflammation and oxidative stress, autonomic nervous system imbalance, and abnormal epigenetic changes.^3^ These mechanistic pathways are also included in the process of developing overweight or obesity, type 2 diabetes, hypertension, and dyslipidemias,^3^ which are metabolic risk factors for cardiovascular disease (CVD).^4^ Therefore, in epidemiologic studies, these cardiometabolic risk factors are often considered mediators or modifiers of associations between air pollution and CVD. Consequently, the investigation of associations between air pollutants and cardiometabolic risk factors has attracted great interest among researchers worldwide.

Many investigators have explored the potential associations of air pollution with cardiometabolic risk factors, but the results remained mixed.^5,6,7,8,9,10^ In addition, most previous studies^5,11,12,13^ focused on only the associations of 1 or 2 cardiometabolic risk factors with air pollution. A gap in the literature persists as to which cardiometabolic risk factors are more sensitive to air pollution. Furthermore, whether cardiometabolic risk factors can mediate or modify the effects of exposure to air pollution on CVD remains unclear. Both issues have been identified as key research needs, because they are important for identifying sensitive participants who are more vulnerable to adverse cardiovascular effects of air pollution.^3^ Such evidence may help researchers, health care professionals, and physicians evaluate the hazardous effects of air pollutants more completely and design targeted strategies for the primary prevention of CVD. Accordingly, our goals were (1) to investigate and compare the associations of long-term exposure to air pollutants with major cardiometabolic risk factors, including overweight or obesity, hypertension, type 2 diabetes, and dyslipidemias; and (2) to explore whether cardiometabolic risk factors mediated or modified the associations between air pollution and CVD. To fulfill these goals, we analyzed data from the 33 Communities Chinese Health Study (33CCHS).

## Methods

### Population

**Figure 1.**
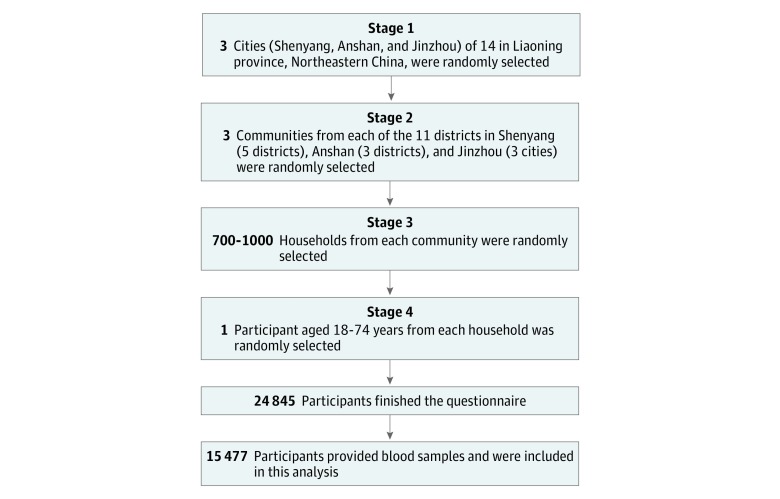
Sampling Strategy for the 33 Communities Chinese Health Study Data were collected from questionnaires from April 1 through December 31, 2009.

The 33CCHS is a large, population-based investigation conducted in the Liaoning province of Northeastern China from April 1 through December 31, 2009. The aim of the 33CCHS was to evaluate the associations of long-term exposure to ambient air pollution with CVD and its metabolic risk factors. The design of the 33CCHS study has been described elsewhere.^11,12,13^ Briefly, using a random number generator, we implemented a 4-stage cluster and random sampling method to recruit study participants (aged 18-74 years) from 33 communities in the cities of Shenyang, Anshan, and Jinzhou (Figure 1). Individuals who lived in the study area for fewer than 5 years were excluded. A total of 28 830 eligible participants were invited to participate in our study. We administered a standardized questionnaire to collect data on sociodemographic, socioeconomic, and lifestyle factors. However, 3985 participants did not complete the survey, leaving 24 845 participants (response rate, 86.2%). We further excluded 9368 individuals who did not provide blood samples. Finally, data on 15 477 participants (62.3%) were used in this analysis. We followed the Strengthening the Reporting of Observational Studies in Epidemiology (STROBE) reporting guideline reporting guideline for cross-sectional studies. The study protocol was approved by the Human Studies Committee of Sun Yat-sen University, Guangzhou, China. All participants signed written informed consent.

### Exposure Measurement

Daily concentrations of particles with aerodynamic diameter of no greater than 1.0 μm (PM_1.0_) and no greater than 2.5 μm (PM_2.5_) were estimated at a resolution of 0.1° × 0.1° for the 33 communities, using ground-monitored PM_1.0_ and PM_2.5_ data, satellite-derived aerosol optical depth data, land use, meteorology data, and other spatial and temporal information.^12,14^ Briefly, we collected aerosol optical depth data from the moderate-resolution imaging spectroradiometer algorithms Dark Target and Deep Blue and combined them using an inverse variance weighting method. Then, using a generalized additive model, we linked ground-monitored PM_1.0_ and PM_2.5_ data (eMethods 1 in the Supplement) with the data for aerosol optical depth, vegetation, land use, and meteorology and other spatial and temporal variables used to estimate PM concentrations. Finally, we used a 10-fold cross-validation process to test the validity of our risk factors. The results showed that the adjusted *R*^2^ value and root mean squared error for monthly estimated PM_1.0_ were 71% and 13.0 μg/m^3^, respectively; for PM_2.5_, 75% and 15.1 μg/m^3^, respectively.

Concentrations of particles of no greater than 10.0 μm in aerodynamic diameter (PM_10.0_), nitrogen dioxide (NO_2_), sulfur dioxide (SO_2_), and ozone (O_3_) were collected using district-specific air monitoring stations, as detailed elsewhere^11^ (eMethods 2 in the Supplement). Briefly, 1 air-monitoring station in each of the 11 study districts recorded concentrations of the study pollutants per hour according to methods set by the State Environmental Protection Administration of China. We used these data to calculate daily mean concentrations of PM_10.0_, SO_2_, and NO_2,_ and 8-hour mean O_3_ concentrations. The 3-year (2006-2008) mean concentrations of the 6 air pollutants were calculated and assigned to each individual living in the corresponding district or community as the surrogates of exposure.

### CVD Status and CVD Risk Factors Measures

We assessed CVD status according to self-report using a study questionnaire. Participants who answered yes to “Has a physician ever diagnosed you with myocardial infarction, heart failure, coronary heart disease, cerebral thrombosis, cerebral hemorrhage, cerebral embolism, or subarachnoid hemorrhage?” were defined as having CVD.^15^

In accordance with international procedures recommended by the American Heart Association,^16^ trained and certified nurses measured blood pressure using a standardized mercury-column sphygmomanometer. We measured blood pressure 3 times with 2-minute intervals between measurements, and the mean of the 3 measurements was recorded. We defined hypertension as mean systolic blood pressure greater than 140 mm Hg, diastolic blood pressure greater than 90 mm Hg, and/or taking antihypertensive medicine within 2 weeks before the interview.^17^

We measured height and weight according to World Health Organization recommendations^18^ and then calculated body mass index (BMI) as weight in kilograms divided by height in meters squared. Obesity was defined as a BMI of at least 30.0; overweight, 25.0 to 29.9; and normal weight or underweight, less than 25.0.^18^

Fasting venous blood levels of triglycerides and low-density lipoprotein cholesterol (LDL-C) were determined. In addition, we conducted an oral glucose tolerance test on participants who agreed to provide venous blood samples, with glucose measurements at 0 and 2 hours after glucose intake. We determined triglyceride, LDL-C, and glucose levels using an autoanalyzer (Hitachi). Hypertriglyceridemia was defined as triglyceride levels of at least 200 mg/dL (to convert to millimoles per liter, multiply by 0.0113), and hyperbetalipoproteinemia was defined as LDL-C level of at least 160 mg/dL (to convert to millimoles per liter, multiply by 0.0259).^19^ Type 2 diabetes was defined as fasting glucose level of at least 126 mg/dL and/or 2-hour glucose level of at least 200 mg/dL (to convert to millimoles per liter, multiply by 0.0555) and/or intake of antidiabetic medication.^20^

### Covariates

We considered the following covariates: age (in years), sex (men or women), educational levels (no school, primary school, middle school, and junior college or higher), career (government employee, factory worker, farmer, or others), family income (≤5000, 5001-10 000, 10 001-30 000, and ≥30 001 Yuan), alcohol intake (drinker or nondrinker), smoking status (smoker or nonsmoker), controlled diet of low calorie and fat intake (yes or no), sugar-sweetened soft drink consumption (≤1, 2-4, or ≥5 d/wk), regular physical activity (yes or no), family history of CVD (yes or no), and residential greenness (defined by normalized difference vegetation index within a 500-m buffer, calculated as near-infrared minus red [visible] regions divided by near-infrared plus red regions).^21^ We also considered per capita gross domestic product in each district as an area-level covariate.

### Statistical Analysis

Spearman rank correlations were performed to evaluate associations with air pollutants. To assess associations between air pollutants (per 10-μg/m^3^ increase) and cardiometabolic risk factors and CVD, we used generalized linear mixed models to generate odds ratios (ORs) with 95% CIs, in which participants and communities (or districts) were considered as first and second level, respectively.^12,13^ Detailed information on generalized linear mixed models is shown in eMethods 3 in the Supplement. We performed the data analyses using single-pollutant models. However, to control for potential confounding by coexposure to another pollutant(s), we regressed highly correlated air pollutants against each other and used model residuals as covariates. We also adjusted for the potential confounders listed in the Covariates subsection of the Methods section. In addition, we performed stratified and interaction analyses to evaluate effect modification of sex (men vs women), age (≥50 vs <50 years), and family history of CVD (with vs without) on associations between air pollutants and cardiometabolic risk factors.

Further, we used 3 approaches to evaluate whether cardiometabolic risk factors mediated or modified the associations of air pollutants and CVD. First, we applied the PROCESS macro for SAS software (version 2.16.3; SAS Institute) to assess the mediating effects of cardiometabolic risk factors on air pollutants and CVD.^22^ To maximize the available statistical power, we operationalized systolic blood pressure, diastolic blood pressure, BMI, and levels of fasting and 2-hour glucose, triglycerides, and LDL-C as continuous mediators.^22,23^ Second, we performed subgroup analyses according to cardiometabolic risk factor (ie, with vs without) to evaluate its modification on the associations between air pollutants (per 10-μg/m^3^ increase) and CVD and added a cross-product term into the overall model to test for interaction on the multiplicative scale. Third, ORs for CVD were estimated in relation to dichotomous cardiometabolic risk factors (ie, with vs without) and categorical concentrations of air pollutants (high [at or above the mean] vs low [below the mean] levels). The relative excess risk due to interaction was calculated to characterize interaction on the additive scale, with an interaction unequal to 0 indicating significance. All statistical analyses were performed on SAS software (version 9.4; SAS Institute), and a 2-tailed *P* < .05 was considered statistically significant.

## Results

### Descriptive Statistics

Mean (SD) age of the study participants was 45.0 (13.5) years, and nearly half were women (47.3% vs 52.7% men) (Table 1). Most participants were nonsmokers (70.0%), did not drink alcohol (75.4%), and did not exercise regularly (68.1%). In total, 738 individuals (4.8%) reported that they were ever diagnosed with CVD. The prevalence of cardiometabolic risk factors ranged from 8.6% (hyperbetalipoproteinemia) to 40.5% (overweight or obesity). The baseline characteristics were similar (although not the same) between participants included in this analysis and the total 33CCHS participants (eTable 1 in the Supplement).

**Table 1.** Main Characteristics of the Study Participants

Characteristics^a^	Value (N = 15 477)
Age, mean (SD), y	45.0 (13.5)
Sex, No. (%)	
Men	8156 (52.7)
Women	7321 (47.3)
Educational attainment, No. (%)	
Junior college or higher	3579 (23.1)
Middle school	9554 (61.7)
Primary school	1863 (12.0)
No school	481 (3.1)
Career, No. (%)	
Government employee	2900 (18.7)
Factory worker	4996 (32.3)
Farmer	2210 (14.3)
Other	5371 (34.7)
Family income, No. (%)	
≤5000 Yuan/y	1167 (7.5)
5001-10 000 Yuan/y	1977 (12.8)
10 001-30 000 Yuan/y	7869 (50.8)
≥30 001 Yuan/y	4464 (28.8)
Smoking status, No. (%)	
Nonsmoker	10 837 (70.0)
Smoker	4640 (30.0)
Alcohol consumption, No. (%)	
Nondrinker	11 668 (75.4)
Drinker	3809 (24.6)
Regular exercise, No. (%)	
Yes	4932 (31.9)
No	10 545 (68.1)
Controlled diet with low calorie and fat intake, No. (%)	
Yes	3861 (24.9)
No	11 616 (75.1)
Sugar-sweetened soft drink intake, No. (%)	
≤1 d/wk	13 621 (88.0)
2-4 d/wk	1286 (8.3)
≥5 d/wk	570 (3.7)
Family history of CVD, No. (%)	
Yes	3794 (24.5)
No	11 683 (75.5)
Outcome variables, No. (%)	
CVD	738 (4.8)
Hypertension	5314 (34.3)
Type 2 diabetes	1694 (10.9)
Overweight or obesity	6271 (40.5)
Hypertriglyceridemia	3494 (22.6)
Hyperbetalipoproteinemia	1333 (8.6)

Abbreviation: CVD, cardiovascular disease.

^a^ Percentages have been rounded and may not total 100.

Mean (SD) air pollutant concentrations were 66.0 (10.7) μg/m^3^ for PM_1.0_, 82.0 (14.8) μg/m^3^ for PM_2.5_, 123.1 (14.6) μg/m^3^ for PM_10.0_, 54.4 (14.3) μg/m^3^ for SO_2_, 35.3 (5.5) μg/m^3^ for NO_2_, and 49.4 (14.1) μg/m^3^ for O_3_ (eTable 2 in the Supplement). In addition to NO_2_ and SO_2_ (*r* = 0.25 for both), moderate to high correlations were observed between air pollutants (*r* = 0.45-0.84).

### Associations Between Pollutants and Cardiometabolic Risk Factors

The associations between air pollutants and cardiometabolic risk factors varied markedly. We detected the strongest associations for hyperbetalipoproteinemia and the weakest associations for overweight or obesity (Table 2). For example, a 10-μg/m^3^ increase in PM_1.0_ was associated with 36% higher odds of hyperbetalipoproteinemia (OR, 1.36; 95% CI, 1.03-1.78), but with 6% higher odds of overweight or obesity (OR, 1.06; 95% CI, 1.02-1.09). Associations for NO_2_ and PM_1_ showed the strongest associations with nearly all risk factors, whereas SO_2_ (OR for hyperbetalipoproteinemia per 10-μg/m^3^, 0.93 [95% CI, 0.79-1.09]) and O_3_ (OR for hyperbetalipoproteinemia per 10-μg/m^3^, 0.89 [95% CI, 0.74-1.08]) showed the weakest associations (Table 2). Little change occurred when we further adjusted the models for other cardiometabolic risk factors in a sensitivity analysis. In sex-stratified analyses, the associations of pollutants with cardiometabolic risk factors were stronger in men than in women (eg, OR for hypertension with per 10-μg/m^3^ increase in PM_1.0_, 1.15 [95% CI, 1.07-1.23] for men and 1.04 [95% CI, 0.96-1.13] for women), with the exception of hypertriglyceridemia (eFigure 1 in the Supplement). Hyperbetalipoproteinemia showed the strongest associations with most air pollutants in men, whereas hypertriglyceridemia showed the strongest associations with most air pollutants in women. Age-stratified analyses showed stronger associations of air pollution with type 2 diabetes and overweight or obesity in the younger age group than in the older age group (eg, OR for type 2 diabetes with per 10-μg/m^3^ increase in PM_1.0_, 1.12 [95% CI, 1.05-1.18] for the younger group and 1.04 [95% CI, 0.98-1.10] for the older group) (eFigure 2 in the Supplement). In both age groups, hyperbetalipoproteinemia showed the strongest associations with PM_1.0_ and PM_2.5_. In stratified analyses by family history of CVD, associations for air pollutants and cardiometabolic risk factors were generally greater in participants with family history of CVD than those without (eg, ORs for hypertension with per 10-μg/m^3^ increase in PM_1.0_, 1.16 [95% CI, 1.08-1.24] for participants with family history of CVD and 1.10 [95% CI, 1.03-1.18] in participants without family history of CVD) (eFigure 3 in the Supplement). Similarly, hyperbetalipoproteinemia showed the strongest associations with PM_1.0_ and PM_2.5_ in both subgroups.

**Table 2.** Associations of Cardiometabolic Risk Factors and CVD Prevalence With Per 10-μg/m^3^ Increase in Air Pollutants

Air Pollutant	Cardiometabolic Risk Factor, OR (95% CI)^a^					
	Hypertension	Type 2 Diabetes	Overweight/Obesity	Hypertriglyceridemia	Hyperbetalipoproteinemia	CVD
PM_1_	1.12 (1.04-1.20)	1.07 (1.01-1.13)	1.06 (1.02-1.09)	1.03 (0.99-1.08)	1.36 (1.03-1.78)	1.10 (1.02-1.20)
PM_2.5_	1.07 (1.02-1.13)	1.04 (1.00-1.08)	1.04 (1.01-1.06)	1.01 (0.98-1.04)	1.22 (1.00-1.48)	1.07 (1.01-1.14)
PM_10_	1.09 (1.05-1.12)	1.08 (1.03-1.13)	1.04 (1.01-1.06)	1.10 (1.06-1.13)	0.97 (0.81-1.17)	1.03 (0.97-1.10)
SO_2_	1.05 (1.00-1.10)	1.04 (1.00-1.08)	1.01 (0.99-1.04)	1.08 (1.05-1.11)	0.93 (0.79-1.09)	1.08 (1.02-1.14)
NO_2_	1.19 (1.10-1.29)	1.20 (1.08-1.32)	1.07 (1.01-1.14)	1.17 (1.09-1.26)	1.18 (0.75-1.88)	1.17 (1.01-1.35)
O_3_	1.05 (1.00-1.12)	1.04 (0.99-1.09)	1.00 (0.97-1.03)	1.10 (1.07-1.14)	0.89 (0.74-1.08)	1.07 (1.00-1.14)

Abbreviations: CVD, cardiovascular disease; NO_2_, nitrogen dioxide; OR, odds ratio; O_3_, ozone; PM_1.0_, particles with aerodynamic diameter of no greater than 1.0 μm; PM_2.5_, particles with aerodynamic diameter of no greater than 2.5 μm; PM_10.0_, particles with aerodynamic diameter of no greater than 10.0 μm; SO_2_, sulfur dioxide.

^a^ Adjusted for age, sex, smoking status, alcohol consumption, household income, controlled diet of low calorie and fat intake, sugar-sweetened soft drink intake, exercise, career, educational attainment, gross domestic product, greenness level, family history of CVD, and residuals from regression model of highly correlated air pollutants.

### Associations Between Air Pollutants and Cardiometabolic Risk Factors and CVD Prevalence

**Figure 2.**
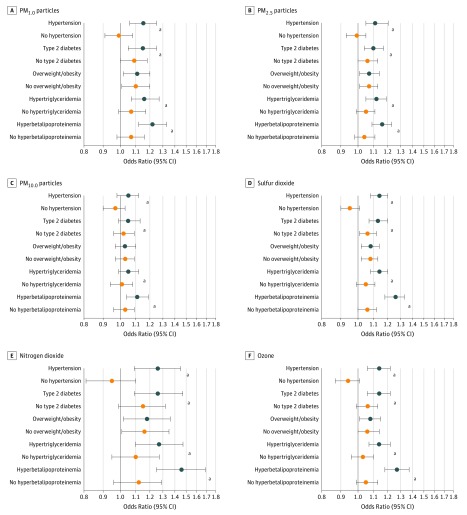
Associations Between Air Pollutants and Cardiovascular Disease Prevalence Stratified by Cardiometabolic Risk Factors Air pollutants include particles with aerodynamic diameter of no more than 1.0 μm (PM_1.0_), particles with aerodynamic diameter of no more than 2.5 μm (PM_2.5_), particles with aerodynamic diameter of no more than 10.0 μm (PM_10.0_), sulfur dioxide (SO_2_), nitrogen dioxide (NO_2_), and ozone (O_3_). The effect estimates (odds ratios and 95% CIs) were scaled to 10 μg/m^3^ in air pollutants and were adjusted for age, sex, smoking status, alcohol consumption, household income, controlled diet of low calorie and fat intake, sugar-sweetened soft drink intake, exercise, career, educational attainment, gross domestic product, greenness level, family history of cardiovascular disease, and residuals from regression model of highly correlated air pollutants. ^a^Interaction is statistically significant (*P* < .05).

**Figure 3.**
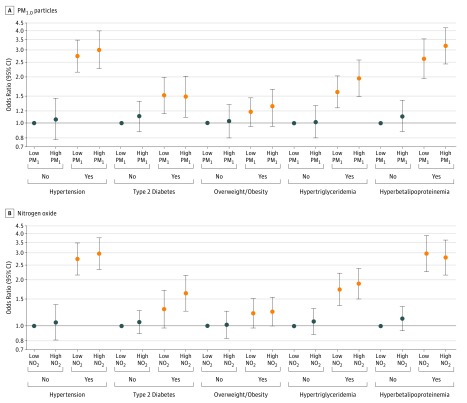
Associations of Dichotomous Air Pollutants and Cardiometabolic Risk Factors With Cardiovascular Disease Prevalence Air pollutants include particles with aerodynamic diameter of no greater than 1.0 μm (PM_1.0_) and nitrogen dioxide (NO_2_). The effect estimates (odds ratios and 95% CIs) were adjusted for age, sex, smoking status, alcohol consumption, household income, controlled diet of low calorie and fat intake, sugar-sweetened soft drink intake, exercise, career, educational attainment, gross domestic product, greenness level, family history of cardiovascular disease, and residuals from regression model of highly correlated air pollutants. Low PM_1.0_ indicates levels of less than 66 μg/m^3^; high PM_1.0_, levels of at least 66 μg/m^3^; low NO_2_, levels of less than 35 μg/m^3^; and high NO_2_, levels of at least 35 μg/m^3^.

All air pollutants (except PM_10.0_) (OR range, 1.07 [95% CI, 1.01-1.14] to 1.17 [95% CI, 1.01-1.35]) (Table 2) and all the studied cardiometabolic risk factors (OR range, 1.23 [95% CI, 1.06-1.43] to 2.79 [95% CI, 2.35-3.31]) (eTable 3 in the Supplement) were significantly associated with increased odds of CVD. In mediation analyses, the associations of air pollutants with CVD were only modestly attributed to cardiometabolic risk factors (except BMI and LDL-C levels), which explains up to 19.4% of the associations (eTable 4 in the Supplement). However, in stratified analyses by cardiometabolic risk factors, the associations between air pollutants and CVD prevalence were consistently higher in participants with hypertension, type 2 diabetes, hypertriglyceridemia, or hyperbetalipoproteinemia than those without these risk factors (eg, ORs for CVD and per 10-μg/m^3^ increase in PM_1.0_, 1.22 [95% CI, 1.12-1.33] in participants with hyperbetalipoproteinemia and 1.07 [95% CI, 0.98-1.16] in participants without hyperbetalipoproteinemia). No effect modification by BMI was observed for all 6 air pollutants (Figure 2). We found similar interaction patterns in subgroup analyses using dichotomous cardiometabolic risk factors and air pollutants in which the participants with both air pollutants at or above mean levels and cardiometabolic conditions generally had the highest odds of CVD relative to the other groups (eg, compared with participants without hyperbetalipoproteinemia and with PM_1.0_ less than mean levels [reference group], OR for CVD was 3.19 [95% CI, 2.43-4.18] in participants with hyperbetalipoproteinemia and PM_1.0_ at or above mean levels) (Figure 3 and eTable 5 in the Supplement).

## Discussion

This study is the first, to our knowledge, to investigate and compare associations between long-term exposure to air pollution and different cardiometabolic risk factors in China. We found associations for hyperbetalipoproteinemia and exposure to air pollutants, especially associated with PM_1_ and NO_2_ levels. Sex, age, and family history of CVD modified the associations, but dyslipidemia still showed the strongest associations with air pollutants (especially with PM_1.0_ and PM_2.5_) in most subgroups. Cardiometabolic risk factors only partially mediated the associations of air pollutants and CVD. However, cardiometabolic risk factors also modified the association such that individuals with these cardiometabolic conditions had a higher prevalence of CVD in association with higher air pollution exposures than individuals without these cardiometabolic conditions.

Few previous studies have simultaneously investigated this set of air pollutants and cardiometabolic risk factors. We are aware of only 2 similar studies, but they were conducted in specific populations (elderly^9^ and adolescents^24^), and their results contradicted ours. The first study^9^ investigated associations of PM_2.5_ with 5 cardiometabolic risk factors in 587 aging US individuals and reported positive associations for elevated fasting blood glucose levels and hypertriglyceridemia, but not for obesity, hypertension, and hypoalphalipoproteinemia. The second study^24^ looked at the air quality index and 6 CVD risk factors in 1413 Iranian adolescents and reported positive associations for elevated fasting blood glucose levels, hypercholesterolemia, and hypertriglyceridemia, but not for elevated blood pressure, hyperbetalipoproteinemia, and hypoalphalipoproteinemia. By comparison, our results included more participants and more air pollutants than the 2 prior studies^9,24^ and provide new evidence of the varied associations between air pollutants and cardiometabolic risk factors in a general population. Three additional previous epidemiologic studies also evaluated air pollutants in association with a panel of cardiometabolic risk factors.^8,9,10^ However, they only reported linear regression coefficients, which cannot be directly compared with ours. In stratified analyses, we detected stronger associations between air pollutants and cardiometabolic risk factors in younger participants and for those with a family history of CVD. Sex also modified the associations, but the pattern of associations was mixed. Although few prior studies assessed the modification effects of family history of CVD, several previous studies evaluated effect modification by sex and age.^6,7,10,25^ However, the results were inconsistent. The reasons for the discrepant results by age and sex are unclear, but may be related to biological (eg, hormonal complement, airway reactivity, and inflammation responses) and lifestyle (eg, outdoor physical activity and smoking) differences.^26^ Nevertheless, consistent with the overall analyses, dyslipidemia showed the strongest associations with PM_1_ and PM_2.5_ in most subgroups, indicating lipid metabolism may be more sensitive to long-term exposure to air pollutants.

Mechanistically, air pollutant exposure could elicit systematic inflammation and oxidative stress, trigger autonomic nervous system imbalance, and cause insulin resistance and abnormal epigenetic changes.^3^ These mechanisms are involved in elevation of blood pressure, glucose levels, and BMI and in lipid metabolism disturbances,^3^ which are consequently capable of instigating CVD events.^3,27^ The statistically significant associations between air pollutants and cardiometabolic risk factors and CVD detected in our study are consistent with these hypotheses.^3^ Two recent experimental studies of gene expression changes after PM_10.0_ exposure found a striking upregulation of many genes encoding the master regulators of lipid and cholesterol synthesis, in addition to upregulated inflammatory genes.^28,29^ This experimental evidence is consistent with our findings that dyslipidemia might be a highly sensitive cardiometabolic risk factor for air pollution exposure. In addition, we detected that PM_1.0_ and NO_2_ showed the strongest associations with CVD. A possible explanation may be that, compared with PM_2.5_ and PM_10.0_, PM_1.0_ has a higher ratio of surface area to mass, which fosters greater access to lung alveoli and the systemic circulation.^30,31^ Also, evidence indicates that PM_1.0_ carries more toxic constituents than PM_2.5_ and PM_10.0_.^32^ Nitrogen dioxide is considered a proxy for traffic-related pollutants and often co-occurs with traffic noise, which has also been linked with adverse cardiovascular health outcomes.^33^ Thus, our NO_2_ effect estimates might have been intensified by the effects of traffic noise.

Cardiometabolic risk factors have long been hypothesized as mediators between air pollutants and CVD.^3,27^ The results of our analyses provide evidence to support these hypotheses. However, only a small part of the associations was explained by these cardiometabolic risk factors. Thus, air pollutants may be associated with CVD primarily via direct effects or joint effects with other mediating factors. As expected, in stratified and interaction analyses, we observed that participants with an existing cardiometabolic risk factor had higher odds of CVD than those without. Thus, participants with hypertension, type 2 diabetes, overweight or obesity, hypertriglyceridemia, and hyperbetalipoproteinemia may be more susceptible to the cardiovascular effects of air pollution than those without cardiometabolic risk factors. Prior epidemiologic studies^34,35,36,37,38,39,40^ that explored cardiometabolic risk factors as effect modifiers of associations of air pollution with CVD primarily focused on short-term exposure or CVD mortality. Only a few published studies to date focused on long-term exposure to air pollution and CVD morbidity, and the results remained mixed.^2,40,41,42,43,44,45^ Nevertheless, our findings were not unexpected, because air pollution exposure and cardiometabolic risk factors are closely associated with increased inflammation, which is also involved in the development of CVD.^3^ Thus, participants with the cardiometabolic risk factors might be more susceptible to the inflammatory effects of air pollutants, leading to a higher CVD prevalence.

### Strengths and Limitations

Our study has several strengths. First, this study had a large sample and a high response rate, offering ample statistical power to detect modest associations and minimize the likelihood for a selection bias. Second, we incorporated a rich set of covariates to control for confounding in the analysis. Third, we used mediation and stratified analyses to help to identify vulnerable subgroups in the study population. Thus, our findings may have important public health implications in identifying sensitive populations who are more susceptible to hazardous effects of air pollution.

Nevertheless, our study also has several limitations. First, the cross-sectional design precludes us from establishing temporality between air pollution and CVD risk markers, in which we cannot be sure if the exposure preceded the outcome or vice versa. Second, the exposure assessment was based on data from districts or communities but not from individuals. This basis might have introduced exposure misclassification with subsequent underestimation of the true underlying association.^46^ Third, although we controlled many potential confounding factors, residual confounding from unmeasured factors (eg, salt intake, consumptions of fruits and vegetables, noise, and walkability) remains possible. In addition, participants with CVD conditions might have modified their lifestyles (eg, diet control, physical activity, and alcohol intake) as a part of a treatment plan; thus, residual confounding owing to covariate misclassification or reverse causality are also possible. Fourth, CVD status was self-reported, and several covariates such as smoking, drinking, and exercise were collected via a questionnaire, so the problem of false-negative findings and errors due to recall bias cannot be ruled out. In addition, some cardiometabolic risk factors (eg, lipid levels) may be more sensitive to temporal changes than others (eg, obesity and type 2 diabetes). However, we only assessed these cardiometabolic risk factors at 1 point, which limited our ability to evaluate the associations with long-term patterns of air pollution exposure and might have misclassified outcomes for some participants. Fifth, the studied air pollutants were highly correlated; thus, we could not evaluate multiple pollutants in single model. However, to accommodate the coexposures, we regressed highly correlated air pollutants against each other and modeled residuals, which were also then adjusted in the main models. Sixth, selection bias is possible because some slight differences occurred in terms of basic characteristics (eg, educational attainment and family income levels) between the included participants and the total 33CCHS participants. Seventh, to maximize our ability to detect modest associations to be confirmed by future studies, we did not correct for type I error inflation, and false-positive associations may have arisen, given the number of exposure-outcome associations evaluated. Thus, our results require replication in a future investigation. Eighth, air pollutant levels in the study sites were very high; thus, our findings may not be readily generalizable to populations in low-pollution areas. However, our results could be relevant to other countries such as India and several African countries, where air quality is declining.

## Conclusions

This study shows comprehensive evidence of associations between long-term exposure to ambient air pollution and cardiometabolic risk factors and CVD prevalence. Dyslipidemia, especially hyperbetalipoproteinemia, showed the strongest associations with air pollutants, especially in relation to PM_1.0_ and NO_2_. Further, participants with cardiometabolic risk factors may be more susceptible to associations of cardiometabolic risk factors and CVD with air pollution. If true, and when the potential mechanisms are better understood, populations with these cardiometabolic risk factors should be prioritized in developing preventive intervention strategies. However, owing to the limitations of our study, further investigations with an improved study design are needed to confirm our findings.

## Notes

Supplement. **eMethods 1.** Explanation for Ground-Monitored Data on PM_1.0_ and PM_2.5_ **eMethods 2.** Explanation for the Air Pollution Data (PM_10.0_, SO_2_, NO_2_, and O_3_) **eMethods 3.** Detailed Information on the 2-Level Binary Logistic Regression Model **eTable 1.** Characteristics of the Study Participants and the Total 33CCHS Participants **eTable 2.** Annual Mean Concentrations and Pairwise Correlations of Air Pollutants **eTable 3.** Associations Between Cardiometabolic Risk Factors and CVD Prevalence **eTable 4.** Proportion Mediated by Cardiometabolic Risk Factors in the Associations of Air Pollutants With CVD Prevalence **eTable 5.** Associations of Dichotomous Air Pollutants and Cardiometabolic Risk Factors With CVD Prevalence **eFigure 1.** Associations Between Air Pollution and Cardiometabolic Risk Factors Stratified by Sex **eFigure 2.** Associations Between Air Pollution and Cardiometabolic Risk Factors Stratified by Age **eFigure 3.** Associations Between Air Pollution and Cardiometabolic Risk Factors Stratified by Family History of Cardiovascular Disease (CVD)
