# Supplementary material for: Association of Long-term Exposure to Ambient Air Pollutants With Risk Factors for Cardiovascular Disease in China
Source: JAMA Netw Open. 2019 Mar 8;2(3):e190318. doi: 10.1001/jamanetworkopen.2019.0318 (PMC6484675; doi:10.1001/jamanetworkopen.2019.0318)
Supplement: Supplement. — eMethods 1. Explanation for Ground-Monitored Data on PM1.0 and PM2.5 eMethods 2. Explanation for the Air Pollution Data (PM10.0, SO2, NO2, and O3) eMethods 3. Detailed Information on the 2-Level Binary Logistic Regression Model eTable 1. Characteristics of the Study Participants and the Total 33CCHS Participants eTable 2. Annual Mean Concentrations and Pairwise Correlations of Air Pollutants eTable 3. Associations Between Cardiometabolic Risk Factors and CVD Prevalence eTable 4. Proportion Mediated by Cardiometabolic Risk Factors in the Associations of Air Pollutants With CVD Prevalence eTable 5. Associations of Dichotomous Air Pollutants and Cardiometabolic Risk Factors With CVD Prevalence eFigure 1. Associations Between Air Pollution and Cardiometabolic Risk Factors Stratified by Sex eFigure 2. Associations Between Air Pollution and Cardiometabolic Risk Factors Stratified by Age eFigure 3. Associations Between Air Pollution and Cardiometabolic Risk Factors Stratified by Family History of Cardiovascular Disease (CVD) [file jamanetwopen-2-e190318-s001.pdf]

Supplementary Online Content

Yang B-Y, Guo Y, Markevych I, et al. Association of long-term exposure to ambient air pollutants with risk factors for cardiovascular disease in China. *JAMA Netw Open*. 2019;2(3):e190318. doi:10.1001/jamanetworkopen.2019.0318

eMethods 1. Explanation for Ground-Monitored Data on $PM_{1.0}$ and $PM_{2.5}$

eMethods 2. Explanation for the Air Pollution Data ($PM_{10.0}$, SO_2, NO_2, and O_3)

eMethods 3. Detailed Information on the 2-Level Binary Logistic Regression Model

eTable 1. Characteristics of the Study Participants and the Total 33CCHS Participants

eTable 2. Annual Mean Concentrations and Pairwise Correlations of Air Pollutants

eTable 3. Associations Between Cardiometabolic Risk Factors and CVD Prevalence

eTable 4. Proportion Mediated by Cardiometabolic Risk Factors in the Associations of Air Pollutants With CVD Prevalence

eTable 5. Associations of Dichotomous Air Pollutants and Cardiometabolic Risk Factors With CVD Prevalence

eFigure 1. Associations Between Air Pollution and Cardiometabolic Risk Factors Stratified by Sex

eFigure 2. Associations Between Air Pollution and Cardiometabolic Risk Factors Stratified by Age

eFigure 3. Associations Between Air Pollution and Cardiometabolic Risk Factors Stratified by Family History of Cardiovascular Disease (CVD)

This supplementary material has been provided by the authors to give readers additional information about their work.

© 2019 Yang B-Y et al. *JAMA Network Open*.

eMethods 1. Explanation for Ground-Monitored Data on $PM_{1.0}$ and $PM_{2.5}$

Ground-monitored PM_1 and $PM_{2.5}$ were obtained from the China Atmosphere Watch Network (CAWNET) of the China Meteorological Administration (CMA). The network consisted of 96 air monitoring stations located across mainland China. Concentrations of PM_1 and $PM_{2.5}$ at all stations were measured with GRIMM 180 Environmental dust monitors (Model 1.108, Grimm Aerosol Technik GmbH, Ainring, Germany). Two quality-control procedures were applied to all PM measurements: a "limit check" and a "climatological check". For the limit check, we evaluated each valid PM measurement to determine whether it fell within its possible limits, otherwise, they were removed. In the climatological check, the median and standard deviation (SD) of hourly PM measurements were calculated at each PM observational site. Any PM values lying outside of more than three SDs from the median PM were removed.

© 2019 Yang B-Y et al. *JAMA Network Open*.

eMethods 2. Explanation for the Air Pollution Data ($PM_{10.0}$, SO_2, NO_2, and O_3)

The operation of the monitoring stations strictly followed quality assurance/quality control (QA/QC) procedures set by the State Environmental Protection Administration of China (SEPAC,1992). The environmental monitoring centers in each of the three study cities conducted regular performance audits and precision checks on the air-monitoring equipment. Quarterly performance audits are conducted to assess data accuracy on PM_{10}, SO_2, NO_2, and O_3 monitoring systems.

1) The calculation method
   The calculation method is performed according to Chinese National standards (GB8170-87). The unit of monitored pollutants is mg/m^3 accurate to the third decimal. The units can also be expressed as μg/m^3, depending on the pollutant's concentration. For concentrations that were too low to be measured, half of the lowest checking limit of the equipment will be used as the measured value.

2) Outliers
   When the measured concentration is too low (e.g. background value), a negative value can be obtained because of the zero drift of the monitor. There is no physical meaning to this value. This negative value can be regarded as a value of "unable to measure."

   For the monitoring station with an automatic calibration system, if equipment zero drift/span drift exceeds the control range during the period of zero/span calibration, the data from the time it becomes out of control until the equipment is recovered should be regarded as invalid data. The data cannot be used statistically.

   The data during the period of zero calibration/span calibration should be regarded as invalid data. It cannot be used statistically, but a flag should be made on these data and the records stored as evidence.

   When values are missing because of a loss of power, any data received by the central control station during the period of the loss of power should be regarded as invalid data. The period of loss of power should be counted at the start of power outage until complete warm-up of equipment. The data cannot be used statistically.

   Because pollutant concentrations change over time and change slowly, there should be no swift change in pollutant concentration in the results of normal monitoring. Either a swift change or no change indicates that there is an equipment problem. The problem should be identified, and the data between the start of problem to recovery should be regarded as outliers. These data cannot be used statistically.

3) Statistics of monitoring data

   One time value

   The central control station uses an average of 15 minutes of pollutant concentrations measured at the branch station as a one-time value. The central control modifies this value and judge whether this value is an outlier using the report software.

   One hour mean value

   At least 75% of the one-time values should be used to calculate the one-hour average mean values. The one-hour average mean value is calculated by averaging all the valid one-time values within one hour.

   Daily average mean

   For PM_{10} at least 12 valid hourly mean values are needed to calculate the daily mean value (using the calendar as the valid time frame), using all available valid hourly mean values. For SO_2 and NO_2 at least 18 hourly mean values everyday are needed to calculate valid daily mean value (using the calendar as the valid time frame). For O_3, at least six hourly concentrations of O_3 per day are needed for calculating the 8-hour average concentration of O_3. All valid hourly mean values are used to calculate the daily mean. (National Environmental Air Quality Standard GB3095-2012)

Monthly mean values are the arithmetic means of all valid daily mean values within the month. Seasonal mean values are the arithmetic means of all valid daily mean values within the season. Yearly mean values are the arithmetic means of all valid daily mean values within the year. District daily mean values are calculated using the monthly mean value, the seasonal mean value, and the yearly mean value from the available air monitoring stations in the district.

© 2019 Yang B-Y et al. *JAMA Network Open*.

eMethods 3. Detailed Information on the 2-Level Binary Logistic Regression Model

At the participant level, we predicted the logit of the prevalence of a cardiovascular disease (CVD) or its risk factors as a function of k covariates (X_1 ….X_k) as follows:

logit [Probability (Y_{ij})] = $\alpha j + \beta_1 X_{1ij} + \ldots + \beta_k X_{1ij} + e_{ij}$ (1)

The variable (Y) in equation 1 is prevalence of CVD or its cardiometabolic risk factor, the subscript j is for study districts (j=1,…, 11) or communities (j=1,…,33), the subscript i is for participants (i=1,..n_j), αj are intercepts at the district or community level, $\beta_1 \ldots \beta_k$ are regression coefficients for covariates, and e_{ij} are the random errors, assumed to have means of zero and constant variance. The αj are random coefficients because they are assumed to vary across districts or communities.

At the district or community level, we regressed the district- specific or community-specific intercepts α_j on the district- specific or community-specific pollutant level (Z_j) to explain variations of α_j, as follows:

$\alpha j = \alpha + \gamma_1 Z_j + u_j$ (2)

Equation 2 predicts CVD or its risk factor prevalence in a district or community by air pollutant concentration Z_j. If γ_1 is positive then districts or communities with higher pollutant levels have a higher prevalence of CVD or its risk factor (adjusting for covariates). Conversely, if γ1 is negative, then the prevalence is lower in districts or communities with a higher pollutant level (adjusting for covariates). The u-terms u_j are random errors at the district or community level, assumed to be independent and have mean of zero and constant variance. These random errors characterize the variation between districts or communities and are assumed to be independent from e_{ij} at the participant level. Note that α, β_1,…, β_k, and γ_1 are not assumed to vary across districts or communities. Therefore, they have no subscript j to indicate to which district or community they belong; they are referred to as fixed effects given that they apply to all districts or communities.

Substituting equation 2 into equation 1 yields a single regression equation:

logit [P (Y_{ij})] = $(\alpha+\gamma_1 Z_j + \beta_1 X_{1ij} + \ldots + \beta_k X_{kij}) + (u_j + e_{ij})$ (3)

The terms in the first and second parentheses in equation 3 are often respectively called the fixed (ordeterministic) and random (or stochastic) parts of the model.

© 2019 Yang B-Y et al. *JAMA Network Open*.

eTable 1. Characteristics of the Study Participants and the Total 33CCHS Participants

Characteristics	Participants (n = 15 477)	Total (n = 24 845)
Age (years, mean ± SD)	45.0 ± 13.5	45.6 ± 13.5
Sex		
Men	8156 (52.7)	12661 (51.0)
Women	7321 (47.3)	12184 (49.0)
Nationality		
Han	14 554 (94.0)	23 470 (94.5)
Others	923 (6.0)	1375 (5.5)
Education		
Junior college or higher	3579 (23.1)	5475 (22.0)
Middle school	9554 (61.7)	14933 (60.1)
Primary school	1863 (12.0)	3446 (13.8)
No school	481 (3.1)	991 (4.0)
Career		
Officials	2900 (18.7)	4443 (17.9)
Workers	4996 (32.3)	7227 (29.1)
Farmers	2210 (14.3)	4880 (19.6)
Others	5371 (34.7)	8295 (33.4)
Family income per year		
≤5000 Yuan	1167 (7.5)	2224 (9.0)
5001-10 000 Yuan	1977 (12.8)	3537 (14.2)
10001-30 000 Yuan	7869 (50.8)	12 348 (49.7)
≥30 001 Yuan	4464 (28.8)	6736 (27.1)
Smoking status		
Nonsmoker	10 837 (70.0)	17 543 (70.6)
Smoker	4640 (30.0)	7302 (29.4)
Alcohol consumption		
Nondrinker	11 668 (75.4)	19 082 (76.8)
Drinking	3809 (24.6)	5763 (23.2)
Exercise		
No	10 545 (68.1)	17 198 (69.2)
Yes	4932 (31.9)	7647 (30.8)
Control diet with low calories and low fat		
No	11 616 (75.1)	18 657 (75.1)
Yes	3861 (24.9)	6188 (24.9)
Sugar-sweetened soft drink		
≤1 day per week	13 621 (88.0)	21 939 (88.3)
2-4 days per week	1286 (8.3)	1974 (8.0)
≥5 days per week	570 (3.7)	932 (3.8)

SD indicates standard deviation.

© 2019 Yang B-Y et al. *JAMA Network Open*.

eTable 2. Annual Mean Concentrations and Pairwise Correlations of Air Pollutants

Exposure	Annual mean concentrations							Spearman correlation coefficient (*P*-value)					
	**Mean (SD)**	**Median**	**Minimum**	**Maximum**	**IQR**	**>WHO (%)**		**PM_1**	**$PM_{2.5}$**	**PM_{10}**	**SO_2**	**NO_2**	**O_3**
PM_1 (μg/m^3)[a]	66.0 (10.7)	62	50	82	15	None[c]		1.00	0.99 (<0.001)	0.73 (0.01)	0.52 (0.10)	0.67 (0.02)	0.47(0.14)
$PM_{2.5}$ (μg/m^3)[a]	82.0 (14.8)	73	64	104	26	100			1.00	0.72 (0.01)	0.51 (0.11)	0.63 (0.04)	0.45 (0.04)
PM_{10} (μg/m^3)[b]	123.1 (14.6)	123	93	145	19	100				1.00	0.81 (0.003)	0.65 (0.03)	0.81 (0.002)
SO_2 (μg/m^3)[b]	54.4 (14.3)	48	36	78	20	100					1.00	0.25 (0.46)	0.84 (0.001)
NO_2 (μg/m^3)[b]	35.3 (5.5)	33	27	45	9	18.2						1.00	0.45 (0.16)
O_3 (μg/m^3)[b]	49.4 (14.1)	50	27	71	22	0.0							1.00

Abbreviations: IQR, interquartile range; NO_2, nitrogen dioxide; O_3, ozone; PM_1, particles with aerodynamic diameter ≤1.0 μm; $PM_{2.5}$, particles with aerodynamic diameter ≤2.5 μm; PM_{10}, particles with aerodynamic diameter ≤10 μm; SO_2, sulfur dioxide; WHO, World Health Organization air quality guidelines (2005).

[a]Based on values from 33 communities.

[b]Based on values from 11 districts.

[c]A guideline for PM_1 has not been proposed by WHO.

© 2019 Yang B-Y et al. *JAMA Network Open*.

eTable 3. Associations Between Cardiometabolic Risk Factors and CVD Prevalence

	OR (95% CI)			
**CVD metabolic risk factors**	**Crude model**	***P*-value**	**Adjusted model[a]**	***P*-value**
Hypertension	4.63 (3.94, 5.43)	<0.001	2.79 (2.35, 3.31)	<0.001
Type 2 diabetes	2.43 (2.02, 2.92)	<0.001	1.44 (1.18, 1.75)	<0.001
Overweight/obesity	1.25 (1.08, 1.46)	0.003	1.23 (1.06, 1.43)	0.007
Hypertriglyceridemia	1.80 (1.54, 2.11)	<0.001	1.77 (1.50, 2.09)	<0.001
Hyperbetalipoproteinemia	3.83 (3.21, 4.58)	<0.001	2.76 (2.28, 3.32)	<0.001

Abbreviations: CI, confidence interval; CVD, cardiovascular disease; OR, odds ratio.

[a]Adjusted by age, gender, career, education, household income, smoking status, alcohol consumption, exercise, controlled food of low calories and low fat, sugar-sweetened soft drink intake, family history of CVD, and gross domestic product, and greenness levels.

© 2019 Yang B-Y et al. *JAMA Network Open*.

eTable 4. Proportion Mediated by Cardiometabolic Risk Factors in the Associations of Air Pollutants With CVD Prevalence

Exposure	Mediator	% mediated	*P* Value
PM_1			
	Systolic blood pressure	5.6	.03
	Diastolic blood pressure	6.5	.01
	Fasting blood glucose	2.1	.06
	2-hour blood glucose	3.1	.03
	Body mass index	2.0	.15
	Triglycerides	0.0	.78
	Low-density lipoprotein cholesterol	1.1	.71
$PM_{2.5}$			
	Systolic blood pressure	5.3	.06
	Diastolic blood pressure	5.3	.03
	Fasting blood glucose	1.5	.08
	2-hour blood glucose	2.9	.10
	Body mass index	1.5	.15
	Triglycerides	0.0	.48
	Low-density lipoprotein cholesterol	0.0	.78
PM_{10}			
	Systolic blood pressure	13.9	.04
	Diastolic blood pressure	19.4	<.001
	Fasting blood glucose	6.3	.06
	2-hour blood glucose	12.5	.002
	Body mass index	0.0	.42
	Triglycerides	18.2	<.001
	Low-density lipoprotein cholesterol	0.0	.78
SO_2			
	Systolic blood pressure	3.9	.09
	Diastolic blood pressure	5.1	.02
	Fasting blood glucose	1.3	.11
	2-hour blood glucose	4.0	.004
	Body mass index	0.0	.73
	Triglycerides	7.6	<.001
	Low-density lipoprotein cholesterol	0.0	.74
NO_2			
	Systolic blood pressure	5.2	.08
	Diastolic blood pressure	8.7	.002
	Fasting blood glucose	2.5	.06
	2-hour blood glucose	4.3	.01
	Body mass index	0.6	.33
	Triglycerides	5.0	.008
	Low-density lipoprotein cholesterol	0.0	.89
O_3			
	Systolic blood pressure	4.2	<.001
	Diastolic blood pressure	6.9	.02
	Fasting blood glucose	1.4	.23
	2-hour blood glucose	4.2	.01
	Body mass index	0.0	.30
	Triglycerides	9.6	<.001
	Low-density lipoprotein cholesterol	0.0	.84

Abbreviations: CVD, cardiovascular disease; NO_2, nitrogen dioxide; O_3, ozone; PM_1, particles with aerodynamic diameter ≤1.0 μm; $PM_{2.5}$, particles with aerodynamic diameter ≤2.5 μm; PM_{10}, particles with aerodynamic diameter ≤10 μm; SO_2, sulfur dioxide.

© 2019 Yang B-Y et al. *JAMA Network Open*.

eTable 5. Associations of Dichotomous Air Pollutants and Cardiometabolic Risk Factors With CVD Prevalence

Cardiometabolic risk factors	Air pollutants	OR (95% CI)	RERI (95% CI)
**$PM_{2.5}$:**			
Hypertension			0.36 (-0.29, 1.01)
No	$PM_{2.5}$ < 82 μg/m^3	Ref.	
No	$PM_{2.5}$ ≥ 82 μg/m^3	1.06 (0.78, 1.45)	
Yes	$PM_{2.5}$ < 82 μg/m^3	2.74 (2.15, 3.48)	
Yes	$PM_{2.5}$ ≥ 82 μg/m^3	3.01 (2.27, 3.99)	
Type 2 diabetes			-0.11 (-0.68, 0.45)
No	$PM_{2.5}$ < 82 μg/m^3	Ref.	
No	$PM_{2.5}$ ≥ 82 μg/m^3	1.11 (0.88, 1.39)	
Yes	$PM_{2.5}$ < 82 μg/m^3	1.52 (1.15, 1.99)	
Yes	$PM_{2.5}$ ≥ 82 μg/m^3	1.48 (1.09, 2.02)	
Overweight/obesity			0.10 (-0.25, 0.45)
No	$PM_{2.5}$ < 82 μg/m^3	Ref.	
No	$PM_{2.5}$ ≥ 82 μg/m^3	1.03 (0.80, 1.32)	
Yes	$PM_{2.5}$ < 82 μg/m^3	1.18 (0.95, 1.46)	
Yes	$PM_{2.5}$ ≥ 82 μg/m^3	1.29 (1.00, 1.66)	
Hypertriglyceridemia			0.41 (-0.09, 0.92)
No	$PM_{2.5}$ < 82 μg/m^3	Ref.	
No	$PM_{2.5}$ ≥ 82 μg/m^3	1.02 (0.80, 1.30)	
Yes	$PM_{2.5}$ < 82 μg/m^3	1.60 (1.26, 2.03)	
Yes	$PM_{2.5}$ ≥ 82 μg/m^3	1.96 (1.49, 2.58)	
Hyperbetalipoproteinemia			0.05 (-1.02, 1.12)
No	$PM_{2.5}$ < 82 μg/m^3	Ref.	
No	$PM_{2.5}$ ≥ 82 μg/m^3	1.11 (0.88, 1.41)	
Yes	$PM_{2.5}$ < 82 μg/m^3	2.63 (1.95, 3.54)	
Yes	$PM_{2.5}$ ≥ 82 μg/m^3	3.19 (2.43, 4.18)	
**PM_{10}:**			
Hypertension			0.26 (-0.30, 0.83)
No	PM_{10} < 123 μg/m^3	Ref.	
No	PM_{10} ≥ 123 μg/m^3	0.87 (0.66, 1.16)	
Yes	PM_{10} < 123 μg/m^3	2.49 (1.91, 3.23)	
Yes	PM_{10} ≥ 123 μg/m^3	2.62 (2.02, 3.39)	
Type 2 diabetes			0.18 (-0.34, 0.69)
No	PM_{10} < 123 μg/m^3	Ref.	
No	PM_{10} ≥ 123 μg/m^3	0.98 (0.81, 1.20)	
Yes	PM_{10} < 123 μg/m^3	1.32 (0.96, 1.81)	
Yes	PM_{10} ≥ 123 μg/m^3	1.46 (1.12, 1.92)	
Overweight/obesity			-0.06 (-0.40, 0.28)
No	PM_{10} < 123 μg/m^3	Ref.	
No	PM_{10} ≥ 123 μg/m^3	0.99 (0.80, 1.24)	
Yes	PM_{10} < 123 μg/m^3	1.30 (1.02, 1.66)	
Yes	PM_{10} ≥ 123 μg/m^3	1.16 (0.92, 1.46)	
Hypertriglyceridemia			-0.18 (-0.74, 0.37)
No	PM_{10} < 123 μg/m^3	Ref.	
No	PM_{10} ≥ 123 μg/m^3	1.04 (0.84, 1.29)	
Yes	PM_{10} < 123 μg/m^3	1.90 (1.45, 2.47)	
Yes	PM_{10} ≥ 123 μg/m^3	1.74 (1.36, 2.23)	
Hyperbetalipoproteinemia			-0.65 (-1.69, 0.38)
No	PM_{10} < 123 μg/m^3	Ref.	
No	PM_{10} ≥ 123 μg/m^3	1.10 (0.90, 1.35)	
Yes	PM_{10} < 123 μg/m^3	3.26 (2.43, 4.38)	
Yes	PM_{10} ≥ 123 μg/m^3	2.66 (2.02, 3.51)	
**SO_2:**			
Hypertension			0.46 (-0.16, 1.07)

© 2019 Yang B-Y et al. *JAMA Network Open*.

No	SO_2 < 54 μg/m^3	Ref.	
No	SO_2 ≥ 54 μg/m^3	1.13 (0.85, 1.49)	
Yes	SO_2 < 54 μg/m^3	2.64 (2.12, 3.30)	
Yes	SO_2 ≥ 54 μg/m^3	3.33 (2.63, 4.21)	
Type 2 diabetes			0.60 (0.03, 1.16)
No	SO_2 < 54 μg/m^3	Ref.	
No	SO_2 ≥ 54 μg/m^3	1.15 (0.96, 1.38)	
Yes	SO_2 < 54 μg/m^3	1.21 (0.92, 1.58)	
Yes	SO_2 ≥ 54 μg/m^3	1.98 (1.50, 2.62)	
Overweight/obesity			-0.09 (-0.45, 0.27)
No	SO_2 < 54 μg/m^3	Ref.	
No	SO_2 ≥ 54 μg/m^3	1.21 (0.98, 1.49)	
Yes	SO_2 < 54 μg/m^3	1.29 (1.06, 1.58)	
Yes	SO_2 ≥ 54 μg/m^3	1.37 (1.09, 1.71)	
Hypertriglyceridemia			-0.20 (-0.76, 0.37)
No	SO_2 < 54 μg/m^3	Ref.	
No	SO_2 ≥ 54 μg/m^3	1.29 (1.06, 1.58)	
Yes	SO_2 < 54 μg/m^3	1.91 (1.53, 2.38)	
Yes	SO_2 ≥ 54 μg/m^3	2.01 (1.58, 2.57)	
Hyperbetalipoproteinemia			-0.19 (-1.27, 0.89)
No	SO_2 < 54 μg/m^3	Ref.	
No	SO_2 ≥ 54 μg/m^3	1.38 (1.14, 1.66)	
Yes	SO_2 < 54 μg/m^3	3.05 (2.40, 3.88)	
Yes	SO_2 ≥ 54 μg/m^3	3.29 (2.42, 4.48)	
**O_3:**			
Hypertension			0.42 (-1.10, 1.93)
No	O_3 < 49 μg/m^3	Ref.	
No	O_3 ≥ 49 μg/m^3	0.97 (0.72, 1.30)	
Yes	O_3 < 49 μg/m^3	2.51 (1.93, 3.28)	
Yes	O_3 ≥ 49 μg/m^3	2.87 (2.20, 3.75)	
Type 2 diabetes			0.48 (-0.04, 0.99)
No	O_3 < 49 μg/m^3	Ref.	
No	O_3 ≥ 49 μg/m^3	1.03 (0.84, 1.27)	
Yes	O_3 < 49 μg/m^3	1.16 (0.84, 1.61)	
Yes	O_3 ≥ 49 μg/m^3	1.65 (1.25, 2.17)	
Overweight/obesity			-0.16 (-0.53, 0.21)
No	O_3 < 49 μg/m^3	Ref.	
No	O_3 ≥ 49 μg/m^3	1.09 (0.87, 1.37)	
Yes	O_3 < 49 μg/m^3	1.35 (1.06, 1.72)	
Yes	O_3 ≥ 49 μg/m^3	1.25 (0.98, 1.59)	
Hypertriglyceridemia			0.07 (-0.48, 0.61)
No	O_3 < 49 μg/m^3	Ref.	
No	O_3 ≥ 49 μg/m^3	1.07 (0.86, 1.34)	
Yes	O_3 < 49 μg/m^3	1.76 (1.34, 2.31)	
Yes	O_3 ≥ 49 μg/m^3	1.88 (1.47, 2.40)	
Hyperbetalipoproteinemia			0.08 (-0.96, 1.11)
No	O_3 < 49 μg/m^3	Ref.	
No	O_3 ≥ 49 μg/m^3	1.21 (0.98, 1.50)	
Yes	O_3 < 49 μg/m^3	2.96 (2.22, 3.96)	
Yes	O_3 ≥ 49 μg/m^3	3.17 (2.38, 4.23)	

Abbreviations: CI, confidence interval; CVD, cardiovascular disease; O_3, ozone; OR, odds ratio; $PM_{2.5}$, particles with aerodynamic diameter ≤2.5 μm; PM_{10}, particles with aerodynamic diameter ≤10 μm; RERI, relative excess risk due to interaction; Ref, reference; SO2, sulfur dioxide. The effect estimates were adjusted for gender, smoking status, alcohol consumption, household income, controlled diet of low calories and low fat, sugar-sweetened soft drink intake, exercise, career, education, gross domestic product, greenness level, family history of cardiovascular disease, and residuals from regression model of highly correlated air pollutants.

© 2019 Yang B-Y et al. *JAMA Network Open*.

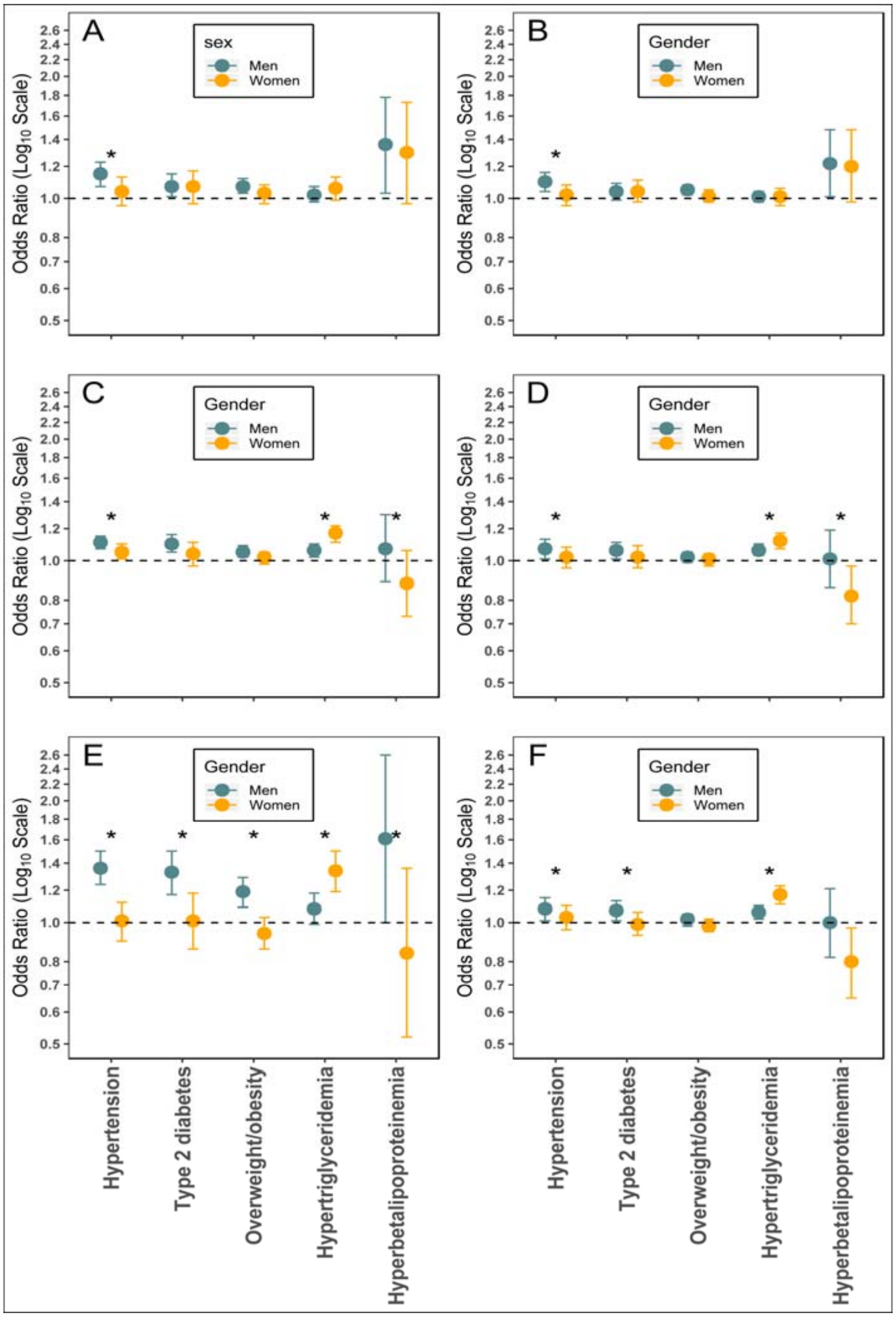

eFigure 1. Associations Between Air Pollution and Cardiometabolic Risk Factors Stratified by Sex

A, for particles with aerodynamic diameter ≤1.0 μm (PM_1); B, for particles with aerodynamic diameter ≤2.5 μm ($PM_{2.5}$); C, for particles with aerodynamic diameter ≤10 μm (PM_{10}); D, for sulfur dioxide (SO_2); E, for nitrogen dioxide (NO_2); F, for ozone (O_3). The effect estimates (odds ratios and 95% confidence intervals) were scaled to 10 μg/m^3 in air pollutants and were adjusted by age, smoking status, alcohol consumption, household income, controlled diet of low calories and low fat, sugar-sweetened soft drink intake, exercise, career, education, gross domestic product, greenness level, family history of cardiovascular disease, and residuals from regression model of highly

© 2019 Yang B-Y et al. *JAMA Network Open*.

correlated air pollutants. (*) indicates $P<0.05$ for the interaction.

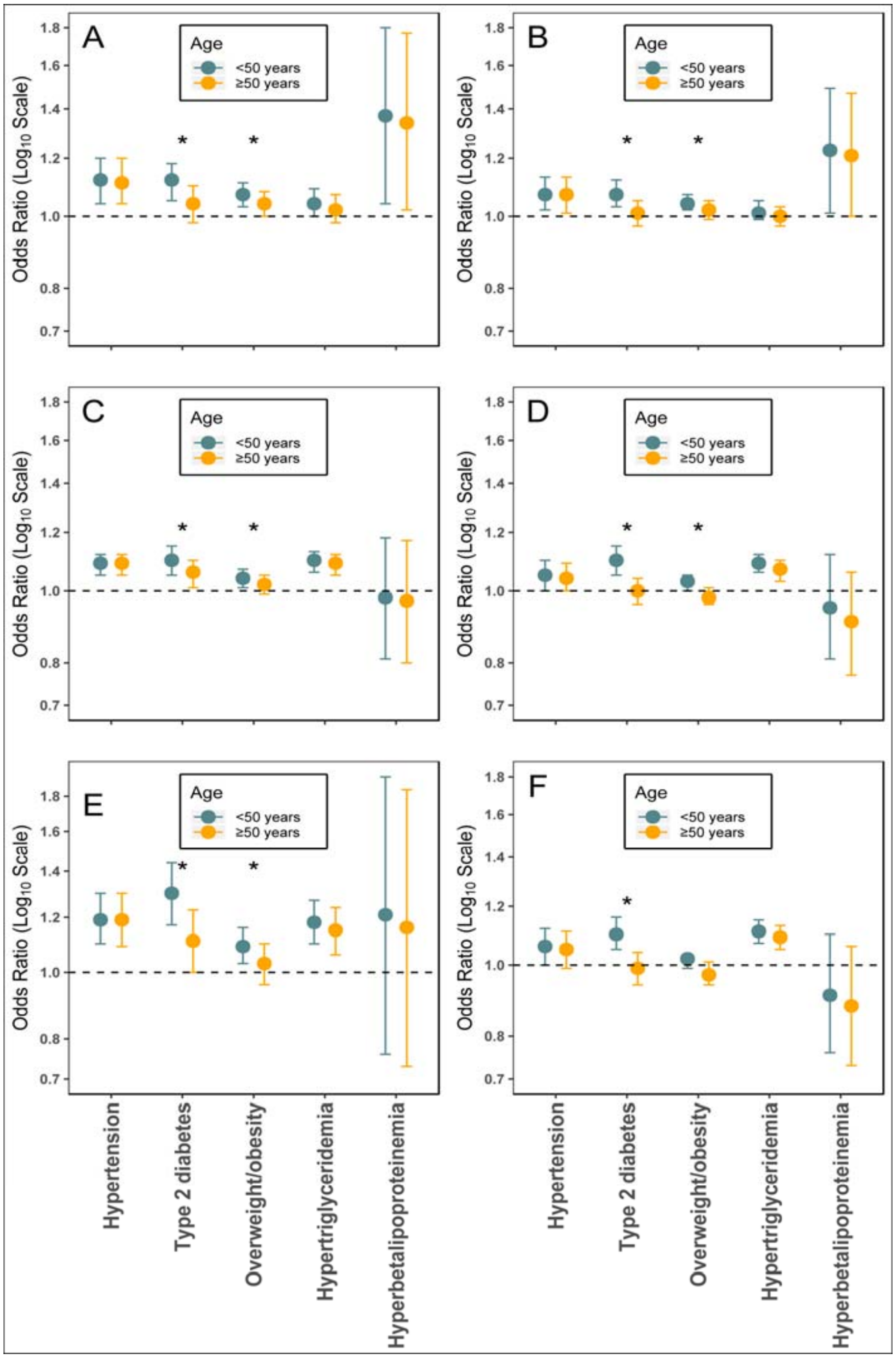

eFigure 2. Associations Between Air Pollution and Cardiometabolic Risk Factors Stratified by Age

A, for particles with aerodynamic diameter ≤ 1.0 μm (PM_1); B, for particles with aerodynamic diameter ≤2.5 μm ($PM_{2.5}$); C, for particles with aerodynamic diameter ≤10 μm (PM_{10}); D, for sulfur dioxide (SO_2); E, for nitrogen dioxide (NO_2); F, for ozone (O_3). The effect estimates (odds ratios and 95% confidence intervals) were scaled to 10 μg/m^3 in air pollutants and were adjusted by gender, smoking status, alcohol

© 2019 Yang B-Y et al. *JAMA Network Open*.

consumption, household income, controlled diet of low calories and low fat, sugar-sweetened soft drink intake, exercise, career, education, gross domestic product, greenness level, family history of cardiovascular disease, and residuals from regression model of highly correlated air pollutants. (*) indicates $P<0.05$ for the interaction.

© 2019 Yang B-Y et al. *JAMA Network Open*.

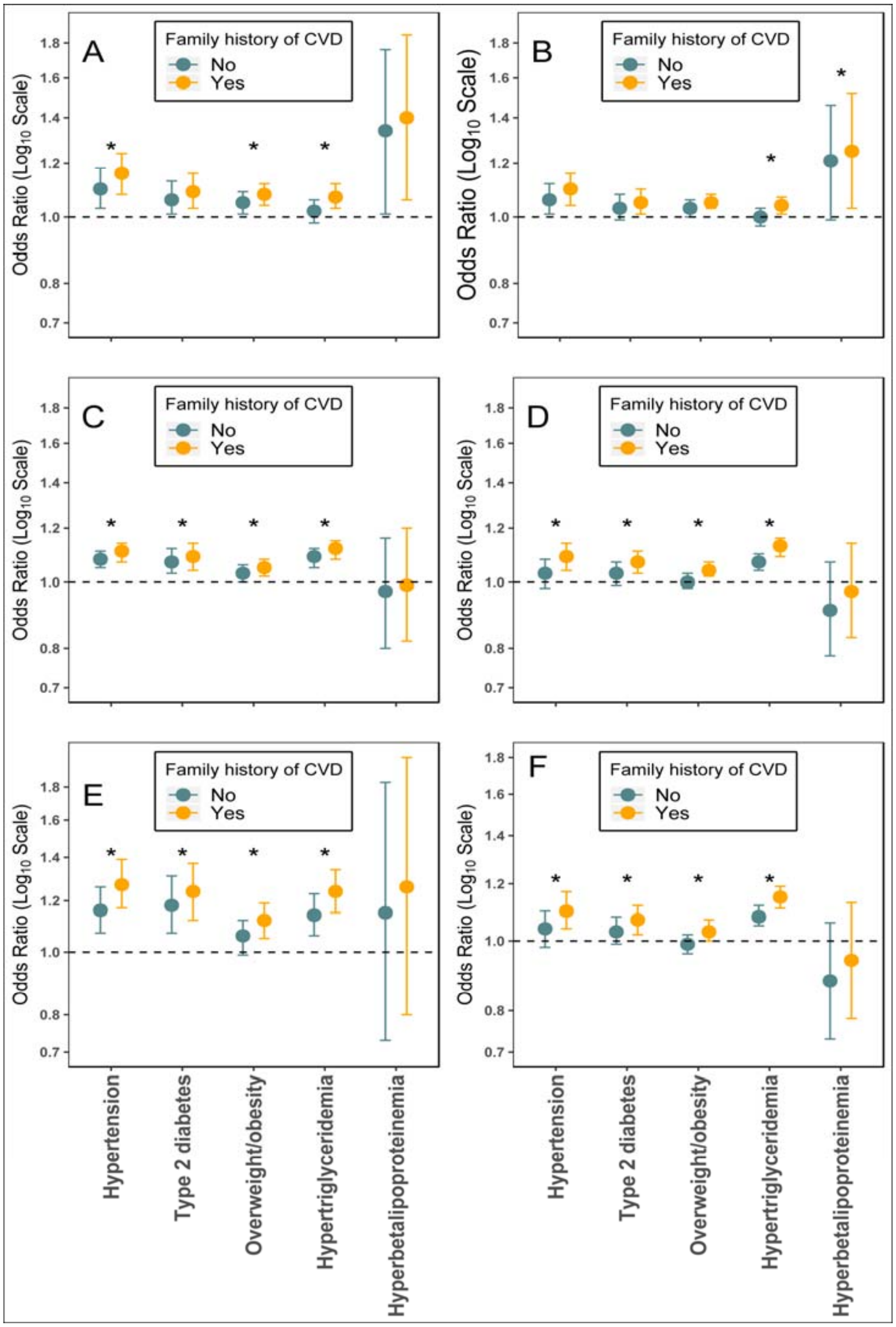

eFigure 3. Associations Between Air Pollution and Cardiomebolic Risk Factors Stratified by Family History of Cardiovascular Disease (CVD)

A, for particles with aerodynamic diameter ≤1.0 μm (PM_1); B, for particles with aerodynamic diameter ≤2.5 μm ($PM_{2.5}$); C, for particles with aerodynamic diameter ≤10 μm (PM_{10}); D, for sulfur dioxide (SO_2); E, for nitrogen dioxide (NO_2); F, for ozone (O_3). The effect estimates (odds ratios and 95% confidence intervals) were scaled to 10 μg/m^3 in air pollutants and were adjusted by age, gender, smoking status, alcohol consumption, household income, controlled diet of low calories and low fat, sugar-sweetened soft drink intake, exercise, career, education, gross domestic product,

© 2019 Yang B-Y et al. *JAMA Network Open*.

greenness level, and residuals from regression model of highly correlated air pollutants. (*) indicates *P*<0.05 for the interaction.

© 2019 Yang B-Y et al. *JAMA Network Open*.
